# The modular cross-synaptic nature of LTP/LTD following on-going neural activity

**DOI:** 10.1186/1471-2202-12-S1-O1

**Published:** 2011-07-18

**Authors:** Alex Loebel, Jean-Vincent Le Bé, Magnus JE Richardson, Andreas Herz, Henry Markram

**Affiliations:** 1 Department Biologie II, LMU, and Bernstein Center Munich, Germany; 2 Brain Mind Institute, Ecole Polytechnique Federale de Lausanne (EPFL), Lausanne, Switzerland; 3 Warwick Systems Biology Centre, University of Warwick, Coventry, UK

## 

While synaptic efficacies are modified continuously by on-going spiking activity, it is yet unclear whether the underlying pre- and post-synaptic processes occur independently, or in accordance. To elucidate the effects of sustained spiking communication on synaptic properties, we patch-clamped paired pyramidal neurons in-vitro at both ends of 12h intervals of spontaneous or glutamate-induced spiking activity. We found that the synaptic efficacies either increased, or decreased, with the ratio between the second and first measurement ranging between 0.08-14. Using quantal and failure analyses we show that this slow form of long-term potentiation and depression is explained by changes in the estimated number of release sites, alongside overall post-synaptic changes that maintain the quantal size per release site. Our findings suggest that sustained neural activity results in matched pre- and post-synaptic modifications, in which elementary modules that span the synaptic cleft are added or subtracted as a function of experience.

